# Exploring the Relationship between the Gut Microbiota and Ageing: A Possible Age Modulator

**DOI:** 10.3390/ijerph20105845

**Published:** 2023-05-17

**Authors:** Juan Salazar, Pablo Durán, María P. Díaz, Maricarmen Chacín, Raquel Santeliz, Edgardo Mengual, Emma Gutiérrez, Xavier León, Andrea Díaz, Marycarlota Bernal, Daniel Escalona, Luis Alberto Parra Hernández, Valmore Bermúdez

**Affiliations:** 1 Endocrine and Metabolic Diseases Research Center, School of Medicine, University of Zulia, Maracaibo 4001, Venezuela; 2 Centro de Investigaciones en Ciencias de la Vida, Universidad Simón Bolívar, Barranquilla 080002, Colombia; 3 Sociedad Internacional de Rejuvenecimiento Facial No Quirúrgico (SIRF), Barranquilla 080002, Colombia; 4 Biological Research Institute “Doctors Orlando Castejon and Haydee V Castejon”, Faculty of Medicine, University of Zulia, Maracaibo 4001, Venezuela; 5 Instituto Ecuatoriano de Seguridad Social, Cuenca 010101, Ecuador; 6 Facultad de Ingenierias, Universidad Simón Bolívar, Cúcuta 540001, Colombia

**Keywords:** gut microbiota, ageing, longevity, centenarian, immune system

## Abstract

The gut microbiota (GM) has been the subject of intense research in recent years. Therefore, numerous factors affecting its composition have been thoroughly examined, and with them, their function and role in the individual’s systems. The gut microbiota’s taxonomical composition dramatically impacts older adults’ health status. In this regard, it could either extend their life expectancy via the modulation of metabolic processes and the immune system or, in the case of dysbiosis, predispose them to age-related diseases, including bowel inflammatory and musculoskeletal diseases and metabolic and neurological disorders. In general, the microbiome of the elderly tends to present taxonomic and functional changes, which can function as a target to modulate the microbiota and improve the health of this population. The GM of centenarians is unique, with the faculty-promoting metabolic pathways capable of preventing and counteracting the different processes associated with age-related diseases. The molecular mechanisms by which the microbiota can exhibit anti-ageing properties are mainly based on anti-inflammatory and antioxidant actions. This review focuses on analysing the current knowledge of gut microbiota characteristics and modifiers, its relationship with ageing, and the GM-modulating approaches to increase life expectancy.

## 1. Introduction

The gut microbiota (GM) comprises a group of bacteria, viruses, archaea, and other microbes present in the gastrointestinal tract (GI), which establishes a symbiotic relationship with its host and directly influences their health status [1,2,3]. Likewise, each individual’s GM is unique, constituted by approximately 100 trillion microorganisms whose singularity is comparable to a fingerprint [4]. In addition, the GM modulates immune [5], cardiovascular, and central systems activity, fulfilling fundamental roles in the human body’s homeostasis [6,7]. 

GM composition and interactions vary according to diverse agents’ interactions throughout life, highlighting the individual’s lifestyle and sociodemographic factors [8,9]. Hence, both its functioning and relationship with the host may be affected by a variety of environmental and biological factors driving anomalies in number and bacterial composition, thereby leading to intestinal dysbiosis (ID), a condition implicated in the development and progression of many metabolic [10], immunological [11], respiratory [12], cardiovascular [13], and neurodegenerative diseases [14], and even the ageing process [15]. 

Due to its correlation with several health parameters, several research groups have sought to determine the implications of gut microbiota in longevity and the ageing process [16,17]. Furthermore, developments in microbiome research have allowed the elucidation of differences in the GM composition of centenarians and the rest of the population, thereby highlighting its importance in the ageing process [18]. However, most research in this field has focused on developing therapeutic tools to modify the GM, providing a window to control the ageing process and promote longevity [16]. In this respect, prebiotics, probiotics, symbiotics, a healthy diet, regular physical activity, and drugs have gained scientific interest in microbial activity regulation.

Under this premise, this review will describe the current knowledge on GM architecture, modifiers, and the relationship between changes in its composition related to ageing and longevity, emphasising the missing link between the taxonomic and functional composition of the microbiota with these processes. Similarly, current scientific evidence regarding ageing management by GM modulation will be presented to clarify the therapeutic properties of diet, prebiotics, probiotics, and physical activity.

## 2. Materials and Methods

A narrative review with an extensive literature search on Scopus, EMBASE, PubMed, ISI Web of Science, and Google Scholar databases, from inception to March 2023, was conducted. The articles recovered for this review were only those in English and Spanish. There were no restrictions according to study type, and only scientific articles from high-impact journals were selected. The terms “dysbiosis”, “microbiota”, “longevity”, “prebiotic”, “ageing”, “ageing”, “probiotic”, and “physical activity” were the keywords used in the search. 

## 3. A General View on the Gut Microbiota: Features, Composition, and Modifiers

The completion of The Human Microbiome Project paved the way for considerable developments in the field of microbiomes and shed some light on its implications for health and disease [19]. Since then, the scientific community has advanced in GM metagenomics, allowing an approximation of functionality and taxonomical composition [20]. Currently, it is recognised that approximately 95% of the microorganisms present in the GM are anaerobic bacteria, which comprise at least more than a thousand types to date [21,22]; the Firmicutes and Bacteroidetes phyla are the most abundant, followed by Actinobacteria, Cyanobacteria, Fusobacteria, Proteobacteria, and Verrucomicrobia [23,24].

The most common genera within Firmicutes are *Clostridium* (95% of Firmicutes), *Lactobacillus*, *Bacillus*, *Enterococcus*, *Eubacterium*, and *Ruminococcus*, whereas, in the case of Bacteroidetes, the most prevalent genera are *Bacteroides*, *Prevotella*, and *Porphyromonas* [25,26]. In addition, more than 3.8 million non-repetitive microbial genes have been characterised, representing a proportion 150 times greater than the complete human genome, of which 99% are of bacterial origin [27]. In addition, it is essential to highlight other relevant microorganisms such as archaea (Methanobrevibacter), fungi (Saccharomyces, Malassezia, and Candida), and some protozoa (Blastocystis) [28]. Along this vein, the characterisation of predominant phyla and genera has inspired the development of enterotypes, which aims to identify patterns of microbiota variation, a concept that remains polemical in the scientific community [29].

Interestingly, the number and diversity of the gut microbiota microorganism vary according to life stage and genetic composition, environmental and disease exposure, and diet. These factors lead a distinctive microbiota development for each individual from birth [30]. However, it is worth noting that factors that may lead to significant changes in specific populations have not been determined yet, as highlighted by Nishijima et al. [31]. Despite its uniqueness, the general features in the microbiomes at different age groups have been identified. In this regard, during the first years of life, there is an expansion in the microbiome’s diversity, which starts declining at age 3, only to stabilise itself at age 5 (acquiring the GM complexity seen in adults), but such GM composition is disrupted during ageing when it loses abundance and diversity [32]. However, the loss in microbe abundance and a reduction in the Bacteroides population have been associated with increased survival chances [19].

**Figure 1:**
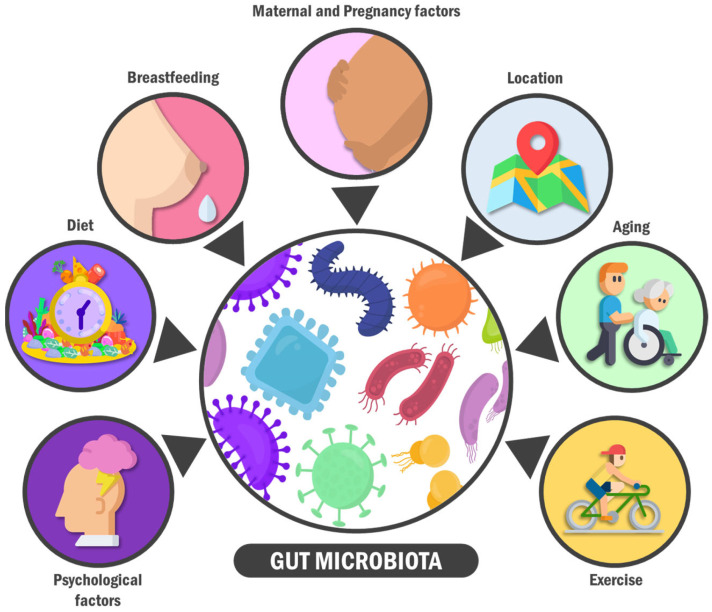
Gut microbiota modifiers. The composition of the intestinal microbiota can be modified by various factors throughout life. The main modifiers include lifestyle, age, the perinatal period and some maternal components, as well as the sociocultural environment and psychological factors of the individual.

On the other hand, certain factors (such as age, psychobiology, and environmental elements) influence individual taxonomic and functional GM variations (Figure 1) [33]. First, gestational conditions, both maternal microbiota and medical history, birth route, and the type of feeding during the first years of life are critical determinants in an infant’s microbiome and GM [34]. For example, it has been considered that the GM composition of babies born at term by vaginal delivery and who are breastfed are optimal for proper child development. Under these conditions, the GM in children tends to be colonised by facultative anaerobic bacteria genera (*Enterobacter*, *Enterococcus*, *Staphylococcus*, and *Streptococcus*), which will subsequently allow the proliferation of strict anaerobic microbes genera such as *Bifidobacterium*, *Bacteroides*, and *Clostridium* [35,36,37].

In turn, preterm babies possess a different GM composition from term babies [38], with a higher proportion of facultative anaerobic and pathogenic bacteria genera, such as Enterococcus and others belonging to the Proteobacteria phylum (*Enterobacter*, *Escherichia*, and *Klebsiella*) [39,40,41], together with reduced levels of strict anaerobic microbes such as *Bifidobacterium*, *Bacteroides*, and *Atopobium*. Such disposition could result from immature organs, feeding, hospital stays, and antibiotic use [42,43,44]. Similarly, birth type is essential to the GM composition of newborns [45]. For example, babies born by vaginal delivery inherit a GM similar to their mother’s vaginal microbiota, so *Lactobacillus*, *Prevotella*, *Escherichia*, *Staphylococcus*, *Bacteroides*, and *Streptococcus* colonies are present [46]. Meanwhile, babies born by caesarean section present a less diverse GM, characterised by acquiring both in-hospital and mother’s skin bacteria (*Staphylococcus*, *Corynebacterium*, *Propionibacterium* spp.). For this reason, there is a lesser proportion of anaerobic bacteria (*Escherichia*, *Shigella*, and *Bacteroides*) [47,48].

Several factors throughout life modify the intestinal microbiota composition. The primary modifiers include lifestyle, age, the perinatal period, and some maternal components, as well as the individual’s sociocultural environment and psychological factors. For example, regarding the perinatal period and some maternal components, in babies born at term by vaginal delivery and breastfed, GM is composed of *Enterobacter*, *Enterococcus*, *Staphylococcus*, *Streptococcus*, *Actinobacteria*, *Firmicutes*, *Lactobacillus*, *Bifidobacterium*, *Bacteroides*, and *Clostridium.* On the other hand, moderate exercise induces positive changes in gut microbiota composition and microbial metabolites produced in the gastrointestinal tract. Furthermore, physical activity frequency is positively associated with a higher gut microbial diversity, shifted toward bacterial species involved in amino acid biosynthesis and carbohydrate metabolism, consequently producing key metabolites such as short-chain fatty acids. Additionally, stress can impact the intestinal barrier’s developmental trajectory and has been associated with increased gut permeability. For example, early-life maternal separation significantly decreases faecal *Lactobacillus* three days post-separation, which correlates with stress-related behaviours.

Nevertheless, term babies born by caesarean section who are exclusively breastfed during their first weeks of life can present GM compositions similar to the GM of babies born by vaginal delivery [49]. Therefore, the infant’s diet is fundamental in developing their GM. In this respect, components in breast milk, such as oligosaccharides and lactoferrin, exhibit prebiotic properties; hence, they can modulate GM composition [50,51]. In addition, breast milk also contains beneficial bacteria that will colonise the infant’s GT [52]. 

In turn, breastfed babies present abundant *Bifidobacterium* in their GMs, which are involved in the metabolism of oligosaccharides from breast milk. In addition, abundance in *Actinobacteria*, *Firmicutes*, *Staphylococcus*, *Bacteroides*, *Streptococcus*, and *Lactobacillus* is also present [53,54]. In contrast, formula-fed babies tend to be frequently colonised by Proteobacteria and *Clostridium* species [55]. However, the weaning period [56] and the mother’s secretory and health status also seem to be determining factors in infants’ GM. Curiously, it has been reported that the GM of preterm babies, who were formula-fed and born by caesarean section, predispose them to suffer from obesity, necrotising enterocolitis, and other diseases, probably due to its interaction with energy metabolism and the maturation of the immune system [57,58,59,60].

Similarly, GM composition and characteristics vary depending on the individual’s dietary habits during infancy and the rest of their lives [61]. Since industrialisation, people have adopted obesogenic habits influencing GM composition and metabolism [62]. For instance, persons with obesity present a high Firmicute over Bacteroidetes rate compared to their lean and overweight counterparts [63]. Furthermore, studies have found a stronger association with Firmicutes phylum species, such as *Blautia hydrogenotorophica*, *Coprococcus catus*, *Eubacterium ventriosum*, *Ruminococcus bromii*, and *Ruminococcus obeum*, in patients with obesity; whereas, lean people have higher proportions of Bacteroides [64]. 

Accordingly, dietary patterns behave as cornerstones in GM composition in all life stages [65]. In this respect, hypercaloric diets (HCD) have been related to increases in Firmicutes and Actinobacteria species and decreases in Bacteroidetes, which agrees with the GM composition observed in obese patients [66]. Similarly, the Western diet englobes high protein, carbohydrate, and fat contents, which is associated with reduced GM diversity characterised by a diminished load of beneficial bacteria, such as Bifidobacterium and Eubacterium, and a more significant proportion of potentially detrimental genera, such as *Shigella* and *Escherichia* [67,68]. Likewise, diets with high caloric restriction are associated with diminished GM variety and enrichment of pathogenic bacteria like *Clostridium difficile*, which negatively influences biliary acid metabolism [69]. In contrast, individuals who follow diets rich in vegetables and fibres, such as Mediterranean dietary patterns, tend to have significant enrichment of *Bifidobacterium*, Bacteroidetes (especially *Prevotella*), and depletion of Firmicutes, which correlates to a lower inflammatory state and a better general health status [70,71].

At the same time, dietary habits correlate to geographic location and GM characteristics. Interestingly, European countries exhibit a North-South gradient concerning child obesity [72]. In this respect, studies have confirmed that this gradient correlates to GM composition, finding that children from northern regions exhibit higher *Bifidobacterium*, *Clostridium*, and *Atopobium* species proportions. In contrast, children from southern regions have higher species diversity, characterised by *Eubacteria*, *Lactobacillus*, and *Bacteroides* richness [73]. On the other hand, African individuals and people from rural Latin America who eat traditional diets rich in vegetables and natural products have healthier and more diverse microbiota compared to GM compositions from areas with Westernised diets (carnivore and fat-diet microbiome) [74,75]. In addition, other factors modify the inter-individual GM taxonomic diversity across all life stages and prominence in some host biological constituents [76], the frequency of physical activity [77], and age [78], which will be addressed in the following sections of this review.

## 4. The Science behind Ageing 

Ageing is a phenomenon consisting of developmental, functional, and morphological changes at the cellular, tissue, and organic levels that occur over time. In mammals, such as humans, age has been considered irreversible due to the “central dogma” that some tissues and cells are irreplaceable. For example, terminally differentiated adult neurons remain in the body throughout an organism’s life and cannot be replaced naturally [79,80,81]. Ageing is the most important risk factor for mortality in humans due to a clear and predictable functional decline, increased frailty, and susceptibility to chronic diseases [80,81,82]. Although its underlying mechanisms have not been completely elucidated yet, a series of common hallmarks have been identified, all of which are predominant during ageing and are common to all human beings; these are classified into three categories: primary, antagonistic, and integrative [83,84]. 

The primary hallmarks include several factors considered detrimental to cellular well-being. This category includes genome instability, telomere shortening, epigenetic alterations, and proteostasis loss [85]. On the other hand, antagonistic hallmarks may be beneficial in narrowly regulated quantities, while their excess produces deleterious effects; these include dysregulation of nutrient sensors, cellular senescence, and mitochondrial dysfunction [86,87]. Lastly, the integrative hallmarks are those phenomena that alter cellular homeostasis; therefore, within this group are found stem cell depletion and altered cell signalling [86,88,89]. Although all these changes contribute to a greater or lesser extent to ageing, the exact triggers and mechanisms of this process remain controversial and are not fully understood [90]. 

In a nutshell, there are two main theories aimed at determining the origins and development of ageing: the programmed or adaptive theories, which state that some “genetic clock” determines the start of ageing in an organism, and the damage or error theories, which explain ageing-related events as consequences of the lack of natural selection as age advances [91,92]. However, these theories and their derivatives could be complementary rather than mutually exclusive. The intrinsic and environmental factors and their reciprocal interaction as determinants of ageing are gaining more recognition among the scientific community [93,94]. Genetics dramatically contribute to the intrinsic factors, where progressive telomere shortening and subsequent genome instability, as well as the expression or suppression of age-related genes such as APOE, FOXO3, or IGF-1R, have been proposed as main mechanisms [95,96,97,98,99,100,101]. In this regard, epigenetic modifications like genome hypomethylation and hypermethylated genomic areas, histone modifications, and chromatin loss have been classically linked to genomic instability, senescence, neoplasia development, and other age-related diseases [102,103,104,105]. 

Other intrinsic elements, such as metabolic pathways and mitochondrial dysfunction, are also involved in ageing through mechanisms that increase the production of reactive oxygen species (ROS) and protein glycosylation. The accumulation of ROS promotes oxidative damage in proteins and lipids, which subsequently leads to alterations in several cellular functions, oxidative membrane stress, misfolded proteins accumulation and the subsequent endoplasmic reticulum stress with decreased autophagy, glycosylation of intracellular proteins, and activation of inflammatory pathways. Moreover, due to its deleterious effects on the extracellular matrix (reduced elasticity in connective tissues), it is thought that ROS build-up could also foster the production of advanced glycation end products (AGEs) and glucose auto-oxidation, which are well-known factors that contribute to ageing and its associated diseases [106,107,108,109,110,111]. On the other hand, lifestyle factors, such as diet, physical activity, socioeconomic status, and habits like smoking, alcohol, and other illicit drugs consumption, through endocrine and immune signalling pathways can induce epigenetic changes, which ultimately promote the expression or suppression of ageing-related genes, having a profound impact on longevity—perhaps even more than the endogenous factor [112,113,114,115,116,117].

In this regard, it is not a matter of debate about the importance of the gut-brain axis. This complex immune-neuro-endocrine network between the gut microbiota and their hosts regulates a myriad of immune, metabolic, and neural functions. Hence, it is a logical candidate as a fine-tuning interface between the external milieu and the inner ambient of our bodies. Moreover, age-related and disease-related deterioration in the gut microbiome of older people reflects overlapping interactive but distinct processes. Thus, resetting gut microbiome-derived signals of ‘unhealthy ageing’ is biologically plausible to slow the ageing process and decrease the prevalence of chronic non-communicable diseases by combating low-grade inflammation and immuno-senescence. 

## 5. Microbiota and Ageing: Finding the Missing Link

### 5.1. Age-Related Microbiome Modifications 

Far from being a static entity, the microbiome suffers various modifications during different life stages of the individual. The transformation of these biocommunities in older adults is particularly evident after considering that when a person reaches an advanced age, they have been exposed to different environmental factors over an extended period [118,119]. However, the microbiome categorisation of older cohorts has proven challenging due to individual variation and the lack of great-scale longitudinal studies analysing GM changes over time.

Beyond these limitations, multiple studies that analysed GM compositions from people of advanced age concluded that there is a general decrease in microorganism diversity and probiotics, together with an increase in opportunistic agents that could be related to age-related chronic diseases [120,121,122,123,124,125,126,127,128,129]. Although the modifications vary according to the specific age group, numerous studies that found differences in the GM composition of elderly groups (ages 99–80 and 79–60) agree on the predominance of the phyla Bacteriodetes and Firmicutes, the first one being more prevalent in the elderly than in younger adults where the phylum Firmicutes is more abundant [19,130,131,132]. 

Similarly, studies have also found decreases in several bacterial groups, including Actinobacteria, certain Ruminococcaceae and Bacteroidaceae members, and *Bifidobacterium*, *Faecalibacterium*, *Eubacterium*, *Bacteroides*, *Clostridium*, and *Oscillospiraceae* genera [133,134,135,136]. On the contrary, numerous microorganisms, mainly opportunistic pathogens and those related to chronic inflammation, increase during ageing, e.g., Cyanobacteria, Lachnospiraceae, Enterobacteriaceae members, and multiple genera such as *Akkermansia*, *Lactobacillus*, *Streptococci*, *Alistipes*, *Prevotella*, *Paraprevotella*, *Helicobacter*, *Eggerthella*, *Coprobacillus*, and *Peptoniphilus* [137,138,139,140,141]. However, it is not adequate to state that the GM of elderly persons loses its diversity and richness in direct proportion to age. Multiple clinical trials indicate general biodiversity increases that surpass that of younger adults [17,142]. In contrast to younger persons, older adults and centenarians present a depletion in *Faecalibacterium prausnitzii* and *Eubacterium rectale* and a more enriched population of *Methanobrevibacter smithii*, *Ruminococceae*, *Proteobacteria*, *Anaerotruncus colihominus*, *Bifidobacterium*, *Porphyromonaceae*, *Rikenellaceae*, *Christensenellaceae*, *Escherichia*, and *Roseburia*; centenarians also have a greater abundance of *Bacteroidetes* compared to younger adults and younger older adults [142,143,144,145,146]. 

In addition to the GM taxonomic changes in the elderly, consequent functional modifications have also been observed [147,148]. For instance, GM from centenarians exhibits increased activity in the phosphatidyl-inositol pathway, glycosphingolipid biosynthesis, N-glycans, and short-chain fatty acids (SCFAs) fermentation. It is also characterised by higher levels of γ-aminobutyric acid (GABA), hydroxybenzoate, polyalkylene glycol (PAG), phenylalanyl methyl chloride (PCS), and 4-imidazole acid. In addition, younger elderly GM shows decreased saccharolytic capacity and low levels of acetate, propionate, butyrate, tryptophan, indole, and nicotinamide [120,149,150]. Finally, reprogramming the saccharolytic metabolic profile to a proteolytic profile is frequently observed due to decreased bacterial species associated with carbohydrate fermentation. Low levels of allantoin, guanosine, butyric, and isovaleric acid, and Mitochondrial Pyruvate Carrier 1 (MCP1) expression [135,151] are also found in young elderly. 

Microbiome experiences modifications not only in response to ageing but also to diverse exogenous stimuli such as dietary patterns, medications, and various pathologies [152]. In this sense, it is essential to highlight the impact of drugs influencing microbiome composition (directly or indirectly). In this category, antibiotics and proton-pump inhibitors (PPIs) are modifiers of the diversity and proportions of microorganisms within the microbiome in different organs by significantly decreasing acid secretion [153]. Long-term administration of PIPs may result in significant changes across the digestive system, such as increased quantities of Firmicutes and decreased Bacteroidetes and Proteobacteria in the oesophagus. At the same time, the gastric microbiota showcases increments in the abundance of Streptococcaceae predominantly, followed by Prevotellaceae, Campylobacteraceae, and Leptotrichiaceae. Similarly, chronic treatments with PIPs are linked to bacterial overgrowth of microorganisms such as Streptococcus, Staphylococcus, Escherichia, Salmonella, Campylobacter, Enterobacteriaceae Klebsiella, Lactobacillus, Veillonela, and Clostridium in the small intestine and colon. Due to dysbiosis, the organism is prone to suffer GI infections, small intestinal bacterial overgrowth (SIBO), chronic inflammation, malabsorption and even oesophagal, gastric, or colorectal cancer [154]. 

Furthermore, the microbiome association with antibiotics has been well documented and varies according to the specific drug and treatment duration. For instance, clinical trials using 500 mg of ciprofloxacin two times a day for seven days have resulted in minor changes (73% similarity to the original state). These positive results are similar to other antibiotics, such as cefazolin, which did not drastically alter the microbiota. However, treatments with drugs such as amoxicillin/clavulanic acid or clindamycin resulted in major changes to all bacterial groups (Clostridium, Enterobacteriaceae, Bifidobacterium, and Bacteroides). Dysbiotic changes induced by antibiotics are generally temporary; nevertheless, in some cases, the consequences can transcend and predispose the individual to pathologies such as obesity, GI perturbations, or bacterial resistance [155,156,157]. 

In this context, microbiome changes might result from different pathologic conditions, from GI disorders to autoimmune, respiratory, or neurodegenerative diseases. Among the autoimmune pathologies such as Type 1 diabetes and rheumatoid arthritis that have displayed reduced bacterial diversity, individuals with RA possess an increase in *Prevotella*, *Porphyromonas gingivalis*, and Bacteroides, as well as reduced Clostridia, Lachnospiraceae, and *Bacteroides fragilis*; meanwhile, DM1 patients present lower levels of *Bacteroides*, *Bifidobacterium*, and *Lactobacillus*, and increased abundance of *Bacteroides ovatus* [158]. A clinical trial carried out by XXX has revealed a higher abundance of Bifidobacterium genus, which was associated with a higher T1D risk (OR: 1.605; 95% CI, 1.339–1.922), implying a causal relationship between gut microbiota alterations and autoimmune diseases [159]. 

Another condition notoriously associated with dysbiosis is obesity. Multiple studies have demonstrated microbiome modifications in these patients, such as increased Firmicutes, Prevotella, Bacteroidetes, Lactobacillus, Faecalibacterium, or *Methanobrevibacter smithii* [155,160]. Moreover, the knowledge regarding microbiota alterations and respiratory diseases is a subject of the current controversy. Nevertheless, multiple studies show less diverse microbiota than healthy individuals. In this sense, asthma may impact the microbiome within the respiratory tract, causing reduced levels of Bacteroides, Lactobacillus, Clostridium, and Bifidobacterium in these patients [161]. 

Liver diseases have also been linked to bacterial dysbiosis through increased intestinal permeability and bacterial translocation that could potentially facilitate the arrival of bacterial metabolites to the liver, impairing bile acid metabolism, gut dysmotility, and inflammation. Major alterations comprised the phyla Bacteroidetes and Firmicutes, decreased Bifidobacterium, Lactobacillus, Enterococcus, and Clostridiales, and increased Proteobacteria, Prevotella, Faecalibacterium, Klebsiella, Proteus, Streptococcus, and Veillonella, among others [162].

Finally, microbiome dysbiosis represents an increasingly relevant field in neurodegenerative and psychiatric disorders [163]. From diseases such as Alzheimer’s, attention deficit hyperactivity disorder (ADHD), amyotrophic lateral sclerosis (ALS), autism spectrum disorder (ASD), anorexia nervosa (AN), bipolar disorder (BD), eating disorder (ED), generalised anxiety disorder (GAD), major depressive disorder (MDD), multiple sclerosis (MS), obsessive-compulsive disorder (OCD), Parkinson’s disease (PD), posttraumatic stress disorder (PTSD), spinal cord injury (SCI), to schizophrenia, the modifications differ between conditions, displaying either a beneficial or detrimental role as well as, specific microbial changes in each case [164,165].

### 5.2. Microbiota: An Age-Modulator Agent?

**Figure 2:**
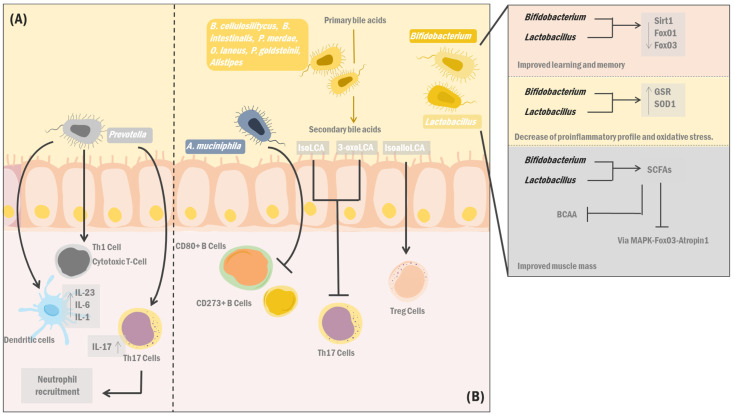
Implications for healthy ageing related to gut microbiota. GM is capable of influencing healthy aging as well as inducing age-related diseases. On the one hand (**A**) Prevotella species have the faculty to induce dendritic cell activity and the release of proinflammatory cytokines (IL23, IL-6 and IL-1). However, there are bacterial strains associated with longevity (**B**), including: A. muciniphila, which de-creases activation of CD80+ and CD273+ B cells in Peyer’s patches, accompanied by attenuation of immu-ne-associated processes. They modulate the decrease of the proinflammatory profile and oxidative stress through the positive regulation of antioxidant enzyme.

In recent years, scientific evidence has shown the possibility of delaying ageing by manipulating the regulatory pathways involved in its bidirectional relationship with the GM. It is known that there is a relationship between intestinal dysbiosis and multiple age-related diseases, such as inflammatory bowel and musculoskeletal diseases and neurological conditions (Figure 2). Nevertheless, there are beneficial bacteria that, rather than deleterious consequences of ageing, may contribute to homeostasis maintenance and healthy ageing. 

GM influences healthy ageing as well as inducing age-related diseases. On the one hand, (A) Prevotella species have the faculty of inducing dendritic cell activity and the release of proinflammatory cytokines (IL23, IL-6 and IL-1), probably through the binding of LPS to TLR2, and in turn mediate the activation of Th1 and cytotoxic T lymphocytes, as well as the modulation of neutrophil recruitment by Th17 cells, which is negatively associated with healthy ageing. On the other hand, bacterial strains associated with longevity (B), including *A. muciniphila*, decreases activation of CD80+ and CD273+ B cells in Peyer’s patches, accompanied by attenuation of immune-associated processes. Likewise, Lactobacillus and Bifidobacterium strains cause the down-regulation of Sirtuin1 (Sirt1), Forkheag box O1 (FoxO1), and FoxO3, improving learning and memory. Furthermore, they modulate a decrease in proinflammatory and oxidative stress profiles by stimulating antioxidant enzymes such as GSR and SOD1 and decreasing TNF, IL-6, and IL-1b expression. Moreover, these bacteria are potent SCFA synthesisers inhibiting the MAPK-Fox03-Atrogin1 pathway and BCAA catabolism. Finally, Alistipes, *Bacteroides cellulosilitycus*, *Bacteroides intestinalis*, *Parabacteroides merdae*, *Parabacteroides goldsteinii*, and *Odoribacter laneus* possess enzymes producing secondary bile acids such as iso-, 3-oxo-, and isoalolithocholic acid; the first two suppress T helper 17 cells effectively. At the same time, the last one induces Treg cells.

In this vein, *A. muciniphila*, usually abundant during ageing, improves colonic mucus thickness and attenuates processes related to the immune system due to decreased CD80 CD273 B cell activation in Peyer’s patches [166]. In turn, *lactobacillus* strains can improve the individual’s lipid profile by activating the AMP-activated protein kinase (AMPK) and its accelerating effects on energy and lipid metabolism [167]. Moreover, *Lactobacillus* and *Bifidobacterium* strains augment learning and memory capacities by down-regulating neurodegenerative markers such as Sirtuin 1 (Sirt1) and members of the Forkhead box (FOX) family: FOXO1 and FOXO3. Additionally, these bacteria up-regulate genes related to myocyte survival and differentiation, thereby improving muscular function and decreasing the proinflammatory profile and oxidative stress. This phenomenon is the consequence of their capacity to up-regulate antioxidant enzymes such as glutathione disulfide reductase (GSR) and superoxide dismutase (SOD1), together with the reduction in the expression of proinflammatory cytokines such as tumour necrosis factor (TNF), interleukin 6 (IL-6) and IL-1b [168].

On the other hand, emerging studies have associated the abundance of *Prevotella* species with systemic diseases, such as rheumatoid arthritis (RA), certain metabolic disorders, and low-grade inflammation [169]. Possibly, the negative association between longevity and healthy ageing with *Prevotella* is due to the ability of this bacterial strain to induce dendritic cell activity and the release of proinflammatory cytokines (IL-23, IL-6 e IL-1), probably through the recognition of lipopolysaccharides (LPS) by the toll-like receptor 2 (TLR2). Furthermore, this bacterial strain mediates CD4 and CD8 T cell activation and modulates neutrophil recruitment via the secretion of IL-17 by Th17 cells [170]. In contrast, *Prevotella histicola* has shown to be a potent immunomodulatory bacterium, capable of suppressing the release of inflammatory cytokines (IL-2, IL17), monocyte chemoattractant protein 1 (MCP-1) and TNF-α. At the same time, this species could induce the release of anti-inflammatory cytokines such as IL-4 and IL-10, resulting in improved proinflammatory profiles seen in age-related diseases such as AR [171,172].

However, certain age-related musculoskeletal conditions have been associated with decreased GM biodiversity and SCFA production [173]. In this regard, the GM can use amino acids involved in muscle protein synthesis, and therefore, it can also alter bioavailability and the individual’s muscular phenotype. Moreover, preclinical and clinical studies have shown that bacteria from the genus *Sutterella*, which significantly increases in older adults, may have an essential role in muscle mass loss, probably due to its relationship with alterations in vitamin B12 levels [174,175]. In contrast, an adequate GM composition during ageing could positively influence the muscular system. For instance, *Lactobacillus* and *Bifidobacterium* strains can restore age-related muscle loss due to the synthesis of SCFA molecules that inhibit the MAPK-FoxO3-Atrogin1 and branched-chain amino acids pathways [176]. Similarly, enzymes found in the GM increase the bioavailability of amino acids (e.g., leucine) via the breakdown of peptides and polypeptides, leading to the mTOR pathway activation and, subsequently, myofibril synthesis gene expression [177]. However, even though the gut-muscle axis has been proposed, its exact mechanisms remain to be elucidated. 

Curiously, studies on centenarians (individuals over a hundred years old), as a life expectancy extreme model, do not fully support the GM changes associated with ageing. However, centenarians have similar GM compositions to young adults, presenting themselves as an advantage in maintaining homeostasis. Therefore, advances in this field have shed some light on the relationship between the microbiome, healthy ageing, and longevity [178]. In this context, a study collecting faecal samples from centenary individuals identified bacterial species, genes, and pathways that promote the generation of unique secondary bile acid derivatives, such as iso-, 3-oxo-, and isoalolitocolic acid (LCA), through the participation of 5-alpha reductase (5AR) enzymes, 3-b hydroxysteroid dehydrogenase (3bHSDH) and 5-beta reductase (5BR) [177]. The genes encoding these enzymes are presumed to be found in *Alistipes*, *Bacteroides cellulosilitycus*, *Bacteroides intestinalis*, *Parabacteroides merdae*, *Parabacteroides goldsteinii*, and *Odoribacter laneus*. Furthermore, isoalloLCA is known to induce T regulatory cell function, while isoLCA and 3-oxoLCA suppress Th17 cell activity, thereby protecting the host from exaggerated immune responses. In addition, isoalloLCA is a selective, potent antibacterial agent against drug-resistant gram-positive bacteria. Hence, these secondary bile acids may contribute to healthy ageing [179].

In addition, a previous study in Sardinia, Italy, found a large abundance of *Bifidobacterium adolescents*, *Lactobacillus*, and *Escherichia* in the centenary population, which was accompanied by the up-regulation of genes associated with glycolysis and SCFA production [180]. Particularly, SCFAs exert protective functions on the epithelial barrier, supporting the growth of beneficial commensal bacteria and reducing colonisation by opportunistic pathogens; consequently, these compounds regulate gut homeostasis and the immune response. On the other hand, a lower abundance of *F. prausnitzii*, *E. rectale*, and *Ruminococcus sp 5 1 39BFAA* was found, along with a deficiency in genes responsible for carbohydrate degradation (e.g., galactose), resulting in a decrease in the production of simple endogenous carbohydrates. The impact of this finding on the health of the centenary population needs further research [179].

## 6. Therapeutic Approach against Ageing: Influencing the Gut Microbiota to Increase Longevity 

As has been presented, a clear relationship exists between gut microbiota and ageing; therefore, GM is considered a prospective therapeutic target to delay the onset of various ageing processes. Likewise, various methods have been proposed to modify the composition of the GM so that the development of bacterial species that promote organic homeostasis is favoured. 

Therefore, based on clinical and preclinical studies, different strategies have been described to delay ageing and its complications via the consumption of probiotics (living microbes), prebiotics (compounds that favour the growth of beneficial species), and symbiotics (a mixture of the previous two) [181]. In this respect, studies that administered probiotics to canines [182] and mice [183,184,185,186] showed the efficacy of such substances in restoring the immune system homeostasis, reducing the levels of proinflammatory markers (TNF-α, IL-1β, IL-4, IFN-γ), bolster memory, improve both neuronal and synaptic lesions, and finally, contribute to glial cell activation and bone mass increments. 

Nevertheless, human studies have yielded divergent results (Table 1). For example, a meta-analysis compiling data from RCTs involving 564 subjects states that even though the consumption of probiotics increases serum calcium levels, it does not improve markers of bone health [187]. In contrast, Jansson et al. [188] conducted a randomised, double-blind, multicentre placebo study in Sweden involving 249 postmenopausal women for 12 months, where they found three Lactobacillus species prevented bone loss in the lumbar spine. Moreover, a randomised, placebo-controlled study assessed the action of symbiotics in 60 people (ages 65–80) diagnosed with metabolic syndrome (MetS). After two months of symbiotic administration, an anti-inflammatory effect was observed with a reduction in high-sensitivity reactive C protein and TNF- α levels [189]. Similar results were found in a meta-analysis of patients with Diabetes [190]. However, the meta-analysis conducted by Qu et al. [191] determined that there were no statistically significant results regarding the aforementioned inflammatory markers, as well as IL-1 β, IL-6, IL-8, IL-10, and MCP-1. 

In turn, Miller et al. [192] presented the beneficial effects of supplement consumption with *Bifidobacterium animalis* ssp. *Lactis HN019* is an immunomodulator in healthy people since these products increase the anticancer activity of polymorphonuclear and NK cells. It is worth noting that other meta-analyses have highlighted the immunomodulatory effects of probiotics and symbiotics [193,194]. Likewise, another randomised, double-blind, placebo study performed in South Korea on 63 participants over 65 years old found that probiotics had a positive effect on the individuals’ cognitive regions, which resulted from an increase in blood-brain-derived neurotrophic factor (BDNF) levels and a subsequent increase in neuroplasticity [195]. However, several systematic reviews highlight the need for more randomised studies to ascertain the real beneficial effects of probiotics on cognition area, mood, metabolic markers, and ageing-related weakness [196,197]. 

In a different vein, several groups have also looked into the relationship between physical activity, GM, and longevity. In this regard, Zhu et al. [198] conducted a study that compared faecal samples from 897 elderly subjects and 1589 participants aged 18–60 years. Their results found that overweight older subjects who undertook physical activity had diminished alpha diversity, increased phylum Bacteroidetes, and decreased Proteobacteria, Cyanobacteria, and Firmicutes. Even though other studies have reported similar results, they have failed to observe changes in alpha diversity. However, they found increased species associated with improved cardiometabolic health in men and anti-inflammatory effects in women—*Oscillospira* and *Verrucomicrobia*, respectively [199,200]. These correlations could result from their possible role in altering pathways related to the biosynthesis of nucleotides, which are inhibited in older people and reactivated when such individuals engage in regular exercise. However, several systematic reviews raise the need for studies that assess physical activity as a unique factor in GM modification since dietary changes could act as confounding factors. In addition, these reviews also call for clinical trials with better designs and larger samples [201,202].

Accordingly, Ghosh et al. [203] reported that elderly individuals who maintained a Mediterranean diet had a better cognitive function, a low inflammatory state, and a lower risk of age-related degeneration since they favoured the development of a GM with species associated with positive changes, e.g., the production of short-chain fatty acids. In turn, a pilot study on 17 women subjected to the daily consumption of 38 g of blueberries for six weeks showed an increase in the alpha diversity of their GM, especially in species such as *Anaerostipes hadrus*, *F. prausnitzii*, *Ruminococcus bromii*, *E. hallii*, *B. intestinihominis*, *Butyrisimonas virosa*, *B. intestinihominis*, and *F. prausnitzii* [204].

Finally, it is worth noting that even though the clinical evidence in this respect is scarce and has confounding results, targeting the GM may counteract multiple degenerative changes associated with ageing, thereby providing a better quality of life to the older population. However, to achieve this objective, more large-scale controlled clinical trials which assess each possible therapeutic management independently are needed.

## 7. A New Integrative Field: Dysbiosis, Immunosenescence, and the Ageing Gut

In the last 15 years, a new field, cellular senescence, has offered a new terrain of interaction between classical theories of ageing and the functions of the gut microbiota. In biology, senescence encompasses the ageing of cells until they stop dividing, but they do not die, and over time, large amounts of senescent cells (SC) accumulate in tissues [205,206,207]. These cells can be identified by one or more of the following characteristics: increase in cell size, increase in β-galactosidase, cyclin-dependent kinase inhibitors p16INK4a, and p21WAF1 expression. Furthermore, several studies have shown that removing the SC in aged tissues can delay the development and severity of age-related pathologies and improve life expectancy [24]. In addition, it has also been observed that through pharmacological interventions, senescent cells can be selectively eliminated from the body, accompanied by positive changes in life expectancy and a delay in chronic disease development [208,209,210]. From these observations, a key question arises: How can the intestinal microbiota play a role of interest in the production and elimination of senescent cells? The answer to this question may lie in gut dysbiosis and the senescent gut, which also appears to play an important role in developing natural but harmful age-related processes such as immunosenescence and inflammatory ageing [207,211]. 

Immunosenescence describes the age-dependent immune system remodelling that determines alterations in immune response and function affect both innate and adaptive immune responses in in the elderly [120]. For instance, adaptive immunosenescence comprises changes such as a reduced B cell lymphopoiesis, loss of naïve B cells and B cells accumulation, impaired antigen presentation by dendritic cells [212,213,214], CD28− veCD57+ve senescent T cells [215], skewing towards Th17 polarisation, anomalous Th1/Th2 responses [216], increase in CD4+veCD25+veFoxp3+ve T cells [Tregs], and thus, increased cancer risk [217]. On the other hand, innate immunosenescence includes a reduced natural killer cytotoxicity [218], an altered clearance of apoptotic cells, accumulation of non-classical monocytes showing a senescence-associated proinflammatory secretory phenotype [219], reduced neutrophil chemotaxis [220], the decreased bactericidal activity of monocytes and neutrophils [221,222], and altered cytokine production by monocytes and dendritic cells [223]. Thus, immunosenescence is the basis for age-related systemic oxidative and inflammatory stress, which makes the elderly susceptible to many diseases [224]. 

In this context, the association between the gut microbiome and health is on the agenda of several research groups, and some evidence has recently emerged that partly immunosenescence is related to age-associated gut dysbiosis [225]. For example, a direct relationship between the gut microbiota and blood neutrophils immunosenescence has been described through the regulation of TLR and MYD88 signalling pathways [226], as well as changes in the gut microbiota in young people that may be associated with multi-omics variables with paramount value in detecting accelerated ageing by immunosenescence markers [174,227]. 

Intestinal tissue cells, such as epithelial cells and fibroblasts, protect the host’s internal environment from the luminal compartment. At the same time, the intestinal barrier detects changes in nutrients and bacterial metabolites to maintain intestinal immune homeostasis. The progressive increase in age-related SC burden, the chronic environment of cellular senescence, and a senescence-associated secretory phenotype in intestinal tissue may deregulate the normal function of the enterocytes (i.e., barrier function) and ultimately contribute to increased intestinal permeability and being prone to inflammation and infections. In this regard, a recent study showed an age-dependent increase in senescent p16Inka4a+/p21+ cells in several human organs, including colon tissues, suggesting SC accumulation as a function of intestinal ageing throughout the human lifespan [228]. Likewise, one study revealed that intestinal tissue developed strong signatures of cellular senescence by increased expression markers like p16Ink4a, p21Cip1, and SA-β-gal in both WT mice and accelerated ageing model-Ercc1-/Δ mice [229]. Furthermore, an age-dependent increase in DNA damage, cellular senescence (p53/p21WAF1), SASP regulators activation (NFκB, p38MAPK, Cox-2), and metabolic stress was also observed in the intestinal tissue of aged mice, indicating their vulnerability to spontaneous age-related genotoxic stress.

Overall, evidence supports the role of intestinal epithelial cells in exhibiting age-dependent cellular senescence that may contribute to intestinal barrier permeability and altered gastrointestinal homeostasis. In addition, chronic SASP secreted by senescent intestinal cells may promote an inflammatory environment and/or oncogenic transformation that may have deleterious effects on immune activation and the composition of the intestinal microbiome [225,230]. 

## 8. Conclusions

The relationship between GM, ageing, and longevity has been most noticeable in recent years, highlighting its influence and importance in the ageing process. Factors influencing the GM’s inter-individual and taxonomic diversity include host-specific biological components, psychobiological habits, environmental elements, and age. Differences in the GM taxonomic composition between centenary individuals and younger older adults (mostly deficient in the abovementioned processes) have made GM a promising target for anti-ageing therapeutic interventions. 

Beyond its limitations, multiple studies in this field have assessed the consumption of probiotics, prebiotics, and symbiotics, as well as the implementation of physical activity and the Mediterranean diet as therapeutic targets, all of which have GM-modulating effects that could delay the onset of ageing and its complications. It has been suggested that such tools could increase colonic mucus thickness, improve processes related to the immune system, lipid profile, learning and memory, and muscular function, and decrease the proinflammatory profile, oxidative stress, and colonisation by opportunistic species. It is a fact that GM has a strong influence on ageing, so more comprehensive studies are recommended in order to endorse and develop therapeutic tools that allow individuals to extend their life expectancy via healthy ageing. 

## Figures and Tables

**Table 1:** Clinical evidence of gut microbiota improvements on degenerative changes associated with ageing.

Author [Ref]	Treatment	Methodology	Results
Liu et al. [173]	Probiotics, prebiotics, or synbiotics consumption	The study of physiological and molecular changes in aging male Wistar rats aged 8–24 months, analyzing body composition, muscular activity, blood biochemistry, and gut microbiota.	Significant increase in serum calcium levels after consumption of probiotics, prebiotics, or synbiotics compared to the control group. (0.52 mg/dL, 95% CI, *p* = 0.13, I^2^ = 44%). There was no significant difference neither in the bone density of the participants (−0.04 g/cm^2^; 95% CI; *p* = 0.47; I^2^ = 0%) nor in the PTH, OC, and ALP levels, respectively (0.71 pg/mL; 95% CI; *p* = 0.09; I^2^ = 59%), (1.80 ng/mL; 95% CI; *p* = 0.66, I^2^ = 0%), and (−10.64 U/L; 95% CI; *p* = 0.0010; I^2^ = 86%).
Tabrizi et al. [177]	Consumption of probiotics and/or synbiotics	Meta-analysis of 18 RCTs performed in adults with diabetes providing detailed information on inflammatory markers such as IL-6, TNF-α, CRP, and NO after probiotics and/or synbiotics administration compared to a control group.	Decreased levels of TNF-α (SMD = −2.99; 95% CI; *p* = 0.001; I^2^: 96.3) and CRP *p* (SMD = −0.87; 95% CI; *p* < 0.001; I^2^: 90.2) with increased levels of NO (SMD = 1.49; 95% CI; *p* < 0.001; I^2^: 92.1) after supplementing participants with probiotics and/or synbiotics. On the other hand, there was no significant change in IL-6 levels (SMD = −0.65; 95% CI; *p* = 0.306; I^2^: 94.7).
Miller et al. [179]	Consumption of probiotics based on *Bifidobacterium animalis* ssp. lactis HN019	Meta-analysis of 4 controlled trials involving 527 participants supplemented with *Bifidobacterium animalis* ssp. *lactis* HN019 using low-fat milk as a vehicle for 3 to 6 weeks.	An increase in the phagocytic capacity of PMNs was observed (MDS = 0.74; 95% CI; *p* < 0.001) in addition to a moderate increase in the tumoricidal activity of NK cells (MDS = 0.43; 95% CI: 0.08; *p* = 0.02).
Zhong et al. [187]	Physical activity	RCT with 14 female participants divided into a control group and a second group which performed an aerobic and resistance exercise program for eight weeks.	Increase in the phylum Fusobacteria in the control group (F = 5.257, *p* = 0.045). In addition, a significant difference was observed in Betaproteobacteria abundance between both groups (F = 5.149; *p* = 0.047) and a decrease in the Bifidobacteriales order in the control group (F = 7.624, *p* = 0.020).
Ghosh et al. [190]	Nutritional changes	Multicenter RCT with 612 participants (286 men, 326 women) divided into a control group and a second group to which a Mediterranean diet was administered for 12 months, separated into three groups: non-fragile, pre-fragile, and fragile.	A significant decrease in DietNegative Otus was found in all groups. However, dietPositive OTUs increased significantly in the non-fragile group compared to the fragile group. In addition, markers of DietPositive OTUs showed a negative association with levels of inflammatory markers such as IL-17.
Ntemiri et al. [191]	Nutritional changes	A pilot study with 17 women divided into two groups (young and old) who consumed 38 g of freeze-dried cranberry powder daily for six weeks.	The β diversity of the faecal microbiota of older women formed a distinct cluster; however, the sample size, along with its interindividual variability, the trend was considered non-significant (PERMANOVA R2 = 0.03). However, an increase in certain CAGs associated with favourable species was identified.

## Data Availability

Not applicable.

## Abbreviations

GM: gut microbiota; LPS: lipopolysaccharides; TLR2: toll-like receptors 2; Th1: T helper 1 cell; IL: Interleukin; IsoLCA: iso lithocholic acid;3-oxoLCA: 3-oxo lithocholic acid; IsoalloLCA: Isoallo lithocholic acid; SIRT: Sirtuins; Fox01/02: Forkhead Box 01/02; GSR: Glutathione-Disulfide Reductas; SOD1: superoxide dismutase 1; SCFAs: Short-chain fatty acids; BCAA: Branched Chain Amino Acids; MAPK: mitogen-activated protein kinases.

RCTs: randomised clinical trials; PTH: parathyroid hormone; OC: Osteocalcin; ALP: alkaline phosphatase; MetS: metabolic syndrome; IL-6: interleukin-6; TNF-α: tumour necrosis factor-α; CRP: C-reactive protein; NO: nitric oxide; OTU: Operational Taxonomic Units; DietPositive OTU: OTUs with a positive association with adherence scores; DietNegative OTU: OTUs with a negative association with adherence scores; IL-17: interleukin-17; CAG: co-abundance groups.
